# Clinical roles of the aberrantly expressed lncRNAs in lung squamous cell carcinoma: a study based on RNA-sequencing and microarray data mining

**DOI:** 10.18632/oncotarget.18058

**Published:** 2017-05-22

**Authors:** Wen-Jie Chen, Rui-Xue Tang, Rong-Quan He, Dong-Yao Li, Liang Liang, Jiang-Hui Zeng, Xiao-Hua Hu, Jie Ma, Shi-Kang Li, Gang Chen

**Affiliations:** ^1^ Department of Thoracic and Cardiovascular Diseases, First Affiliated Hospital of Guangxi Medical University, Nanning, Guangxi Zhuang Autonomous Region 530021, P. R. China; ^2^ Department of Pathology, First Affiliated Hospital of Guangxi Medical University, Nanning, Guangxi Zhuang Autonomous Region 530021, P. R. China; ^3^ Department of Medical Oncology, First Affiliated Hospital of Guangxi Medical University, Nanning, Guangxi Zhuang Autonomous Region 530021, P. R. China; ^4^ Department of General Surgery, First Affiliated Hospital of Guangxi Medical University (West Branch), Nanning, Guangxi Zhuang Autonomous Region 530021, P. R. China

**Keywords:** lncRNAs, LUSC, biomarker, TCGA, tumorigenesis

## Abstract

Lung squamous cell carcinoma (LUSC) accounts for a significant proportion of lung cancer and there have been few therapeutic alternatives for recurrent LUSC due to the lack of specific driver molecules. To investigate the prospective role of lncRNAs in the tumorigenesis and progression of LUSC, the aberrantly expressed lncRNAs were calculated based on The Cancer Genome Atlas RNA-seq data. Of 7589 lncRNAs with 504 LUSC cases, 884 lncRNAs were identified as being aberrantly expressed (|log2 fold change| >2 and adjusted P<0.05) by DESeq R. The top 10 lncRNAs with the highest diagnostic value were *SFTA1P*,*LINC00968*, *LINC00961*, *LINC01572*,*RP1-78O14.1*, *FENDRR*, *LINC01314*,*LINC01272*, *GATA6-AS1*, and *MIR3945HG*. In addition to the significant roles in the carcinogenesis of LUSC, several lncRNAs also played vital parts in the survival and progression of LUSC. *SFTA1P*, *LINC01272*, *GATA6-AS1* and *MIR3945HG* were closely related to the survival time of LUSC. Furthermore, *LINC01572* and *LINC01314* could distinguish the LUSC at early stage from that at advanced stage. The prospective molecular assessment of key lncRNAs showed that a certain series of genes could be involved in the regulation network. Furthermore, the OncoPrint from cBioPortal indicated that 14% (69/501) LUSC cases with genetic alterations could be obtained, including amplification, deep deletion and mRNA upregulation. More interestingly, the cases with genetic alterations had a poorer survival as compared to those without alterations. Overall, the study propounds a potentiality for interpreting the pathogenesis and development of LUSC with lncRNAs, and provides a novel platform for searching for more capable diagnostic biomarkers for LUSC.

## INTRODUCTION

Lung cancer is the one of the leading causes of cancer deaths in the world. Among all lung cancers, more than 85% are categorized as non-small cell lung cancer (NSCLC), of which lung squamous cell carcinoma (LUSC) accounts for an approximate proportion of 30% [1–6]. Different from lung adenocarcinoma (LUAD), LUSC starts in squamous cells, which are slim, flat cells from histology, which look like fish scales. More importantly, the genetic and epigenetic profiles in the process of tumorigenesis and development vary strikingly between LUAD and LUSC [7–10]. There is a wide range of pivotal molecules verified for LUAD, which leads to great therapeutic improvement for recurrent or unresectable LUAD. Instead, there have been few therapeutic alternatives for recurrent LUSC due to the lack of specific driver molecules or mutations [11–15]. Hence, accurate indicators in the tumorigenesis and development of LUSC are urgently required.

To date, a number of prospective markers for LUSC have been identified; however, the pathogenesis of LUSC is sophisticated. Furthermore, sensitive and specific markers are lacking to identify LUSC in the early stage. Long non-coding RNAs (lncRNAs) have arisen as new master regulators of initiation, progression, and response to specific therapies in a broad variety of solid and hematological neoplasms [16–18]. LncRNAs have also been demonstrated to gain various functions in tumorigenesis of lung cancer. However, most of the studies concerned the general NSCLC, but few focused on LUSC [19]. Thus, identification of LUSC-related lncRNAs, and investigation of their clinical roles and molecular mechanisms are essential for understanding the development and progression of LUSC.

The Cancer Genome Atlas (TCGA) database of LUSC has facilitated the analysis on the high throughput data of various genomic alterations, including non-coding RNAs. The aberrantly expressed genes were identified for LUSC based on TCGA data and those genes that highly mutated were highlighted [20]. The clinical role of the most significantly altered microRNAs was also studied in TCGA LUSC cohort [21]. Most recently, the lncRNA alteration frequencies, but not the expression levels, were investigated by cBioPortal with 504 cases of LUSC, as well as LUAD from TCGA database [22]. Another study also compared the lncRNA profiling in LUAD and LUSC with data from TCGA and Gene Expression Omnibus (GEO). However, the concern of this study was the distinct lncRNA expression pattern between LUAD and LUSC. Furthermore, only the paired tissue samples of RNA-sequencing (RNA-Seq) from TCGA (16 pairs) were analyzed. Even the authors validated their findings with microarray data from GEO (GSE19188), only a small number of cases were involved [23]. Thus, in the current study, we calculated the 884 aberrantly expressed lncRNAs from 7589 lncRNAs in 502 LUSC cases. We further selected the top 10 lncRNAs to evaluate their clinicopathological value and potential mechanism for LUSC.

## RESULTS

### Aberrantly expressed lncRNAs based on TCGA data in LUSC

**Figure 1:**
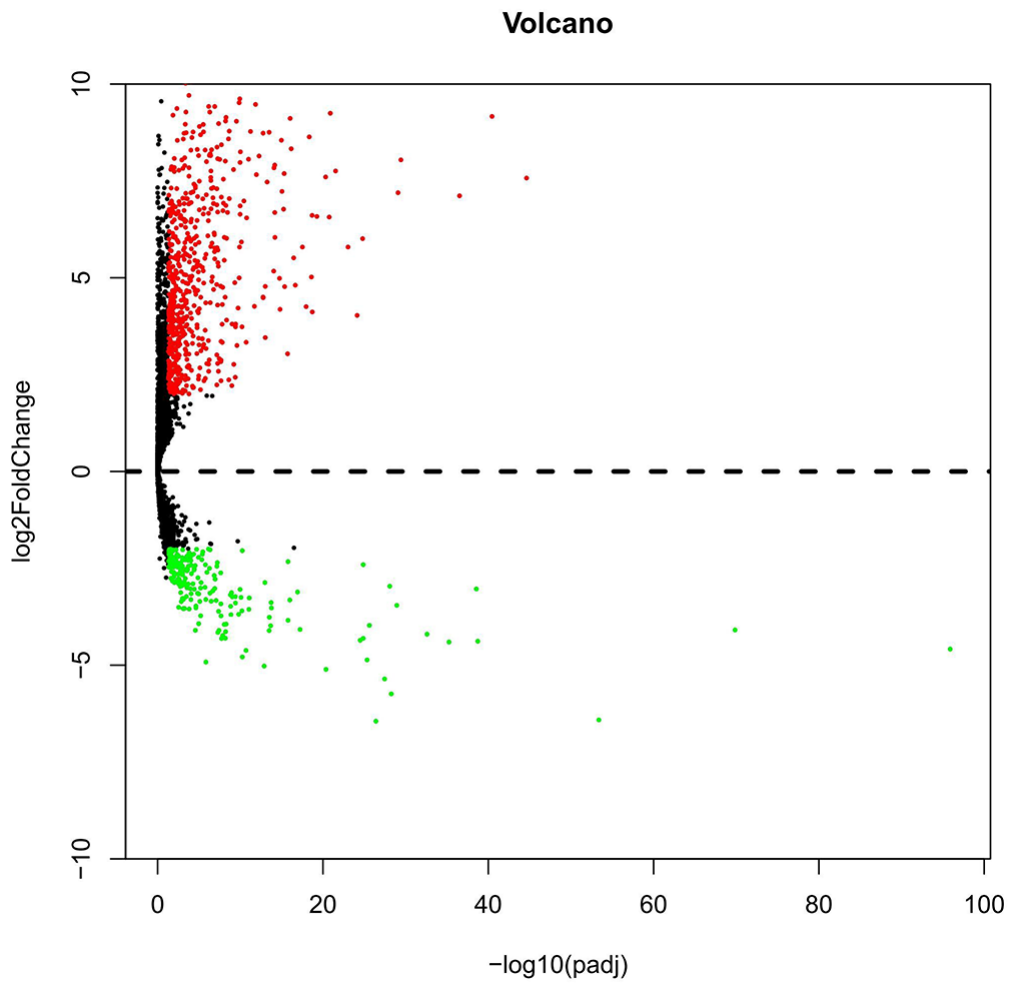
Volcano plot of the aberrantly expressed lncRNAs between LUSC and para-tumorous lung tissues Red dots indicate high expression and green dots indicate low expression of lncRNAs. Black dots show the lncRNAs with expression of |log2FC|<2. The X axis represents an adjusted FDR and the Y axis represents the value of log2FC. Aberrantly expressed lncRNAs were calculated by DESeq R. Altogether, 669 high and 215 low expressed lncRNAs were achieved. This volcano plot was conducted by the ggplot2 package of R language.

The expression level of each lncRNA transformed with log2 was calculated by DESeq R. Following the calculating criteria, we achieved 884 aberrantly expressed lncRNAs (Figure 1) in LUSC, including 669 highly and 215 lowly expressed lncRNAs. All the aberrantly expressed lncRNAs were sent for ROC analysis and we listed the top 75 lncRNAs obtaining over 0.95 for the area under ROC curve (AUC) (Table 1), which demonstrated that these lncRNAs might play essential roles in the occurrence of LUSC and had high diagnostic value for LUSC patients.

**Table 1:** Analysis results of 75 lncRNAs gaining the most significant diagnostic value for LUSC (AUC >0.95)

LncRNA	AUC	FC	Log2FC	P-value	Adjusted P-value
SFTA1P	0.998415	0.041652365	-4.585457785	3.1E-100	1.3E-96
LINC00968	0.997398	0.04726163	-4.403186805	1.18E-38	5.55E-36
LINC00961	0.996585	0.100222391	-3.318723236	1.07E-18	1.01E-16
LINC01572	0.996341	9.626953056	3.267079255	6.51E-07	9.65E-06
RP1-78O14.1	0.995122	0.054413873	-4.199881671	6E-36	2.53E-33
FENDRR	0.994105	0.05863219	-4.09216325	6.64E-74	1.4E-70
LINC01314	0.993983	0.047958874	-4.382058392	2.62E-42	1.85E-39
LINC01272	0.992194	0.122249416	-3.032100523	4.58E-42	2.76E-39
GATA6-AS1	0.991788	0.105033789	-3.251074585	1.82E-12	8.09E-11
MIR3945HG	0.991463	0.050535778	-4.306551063	6.68E-28	1.34E-25
LINC00607	0.990975	0.121229481	-3.044187518	2.48E-12	1.05E-10
PCAT19	0.990772	0.128341572	-2.961939541	2.91E-31	8.19E-29
AC018647.3	0.99069	0.077664175	-3.686606922	3.88E-12	1.56E-10
RP11-108L7.15	0.990284	8.687313032	3.118910023	3.92E-05	0.00038
AC006273.4	0.98817	0.125905728	-2.989584173	1.1E-07	1.86E-06
LINC00702	0.987357	0.115641102	-3.112273834	1.09E-19	1.18E-17
AC109642.1	0.987275	0.091201867	-3.454792838	3.57E-32	1.16E-29
LINC01197	0.986056	0.155580728	-2.684264729	6.63E-09	1.42E-07
CTB-193M12.5	0.985405	5.156248886	2.366321903	7.43E-11	2.45E-09
LINC00511	0.985121	16.33122057	4.029560714	4.14E-27	7.29E-25
RP11-672A2.4	0.984796	0.1040007	-3.265334859	1.55E-13	8.08E-12
RP11-434D9.1	0.982803	0.073633018	-3.763503358	4.61E-16	3.04E-14
LINC00261	0.98256	0.095008585	-3.395798301	1.02E-11	3.81E-10
C14orf132	0.980811	0.188757476	-2.405394311	6.34E-28	1.34E-25
FAM83H-AS1	0.980649	8.212432406	3.037809591	2.14E-18	1.89E-16
Z83851.4	0.979063	6.000254472	2.585023687	3.61E-08	6.63E-07
RP11-532F6.3	0.977275	0.195894607	-2.351850411	2.53E-09	5.97E-08
SLC2A1-AS1	0.976583	10.24741912	3.357188699	7.82E-10	2.12E-08
RP11-161I6.2	0.976319	65.93239774	6.042915643	8.46E-17	6.38E-15
LINC01290	0.975079	0.187150523	-2.417729014	7.79E-06	9.09E-05
RP11-796E10.1	0.974876	54.56086965	5.769794735	5.57E-09	1.23E-07
RP11-513N24.1	0.974429	0.174994013	-2.514622534	3E-06	3.8E-05
RP11-401P9.4	0.974144	0.176841689	-2.499469676	2.8E-08	5.26E-07
AC068831.16	0.974002	35.54345896	5.151512181	8.37E-07	1.19E-05
AC007405.4	0.973778	0.145766461	-2.778269281	4.91E-09	1.09E-07
LINC00472	0.973494	0.215066069	-2.217148164	7.91E-07	1.14E-05
OGFRP1	0.973453	6.108043922	2.610710436	6.7E-06	7.99E-05
RP5-1159O4.2	0.973006	0.207307461	-2.270156058	2.37E-05	0.000245
RP11-560J1.2	0.972823	6.169801561	2.625224089	0.000237	0.001837
CTD-2527I21.15	0.972193	97.72416271	6.610643414	1.59E-21	2.03E-19
RP11-540A21.2	0.972112	6.673744623	2.738496482	1.68E-05	0.000178
CASC9	0.971827	190.5530192	7.574048657	2.21E-48	2.33E-45
RP11-12G12.7	0.971461	5.065939834	2.340829943	8.1E-10	2.15E-08
RP11-613D13.8	0.971014	0.069766914	-3.841313162	1.81E-18	1.66E-16
RP11-245D16.4	0.970729	6.489351573	2.698074329	2.55E-05	0.000261
RP11-473M20.9	0.970567	0.227433967	-2.13648036	2.47E-07	3.9E-06
RP4-758J18.13	0.970323	4.008078031	2.002910596	1.49E-05	0.000162
LINC00519	0.970262	64.45033831	6.010116026	8.24E-28	1.58E-25
RP11-435O5.2	0.968209	4.229037176	2.080329243	4.79E-05	0.00045
RP11-396C23.2	0.967436	8.242877049	3.043147976	1.3E-06	1.77E-05
RP11-284N8.3	0.966257	0.199304014	-2.326957327	1.85E-18	1.66E-16
RP11-236L14.2	0.966095	0.205476098	-2.28295751	4.32E-05	0.000413
PVT1	0.965851	5.393469549	2.431213639	1.1E-11	4.05E-10
AC005537.2	0.96457	36.0538193	5.172080192	1.28E-16	9.04E-15
AC006273.5	0.960363	0.163091113	-2.616249925	9.01E-10	2.33E-08
CTD-2626G11.2	0.959915	0.137024821	-2.86749085	1.73E-15	1.03E-13
CTD-2245E15.3	0.95955	0.188508673	-2.40729719	9.76E-08	1.67E-06
RP11-344B5.2	0.959062	0.248593902	-2.008137185	1.43E-06	1.93E-05
RP11-624L4.1	0.958899	13.3036348	3.733748566	1.33E-12	6.18E-11
CTA-989H11.1	0.956907	5.557899999	2.474539877	4.67E-05	0.000439
RP11-353N14.2	0.956785	15.46687569	3.951109896	6.61E-06	7.91E-05
CARMN	0.955728	0.249734448	-2.001533255	3.89E-08	7.05E-07
AC006129.1	0.955403	0.174046529	-2.522455054	3.18E-06	3.99E-05
RP11-776H12.1	0.955322	55.44550471	5.792998592	2.66E-20	3.03E-18
RP11-244M2.1	0.955078	27.41409845	4.776846124	1.56E-15	9.48E-14
RP13-463N16.6	0.954468	93.40291145	6.545395616	3.44E-13	1.71E-11
RP11-546J1.1	0.953899	5.824459136	2.542124086	0.003303	0.01729
MIR100HG	0.953777	0.20365128	-2.295827214	1.89E-05	0.0002
RP11-1038A11.3	0.95337	27.31391595	4.77156426	4.97E-18	4.28E-16
RP11-429J17.7	0.952801	5.763401812	2.526920605	0.000358	0.002668
RP11-357P18.2	0.951947	0.123202366	-3.020898138	5.65E-09	1.24E-07
RP5-899E9.1	0.951825	0.246349573	-2.021221126	0.000107	0.000915
RP4-616B8.5	0.950524	6.546508194	2.710725601	0.000894	0.005807
LINC00924	0.950159	0.185515247	-2.430390334	2.2E-06	2.86E-05
RP11-7F17.3	0.950037	0.203290027	-2.298388653	8.14E-06	9.4E-05

FC: fold change

### Clinical value of the top 10 aberrantly expressed lncRNAs in LUSC

**Figure 2:**
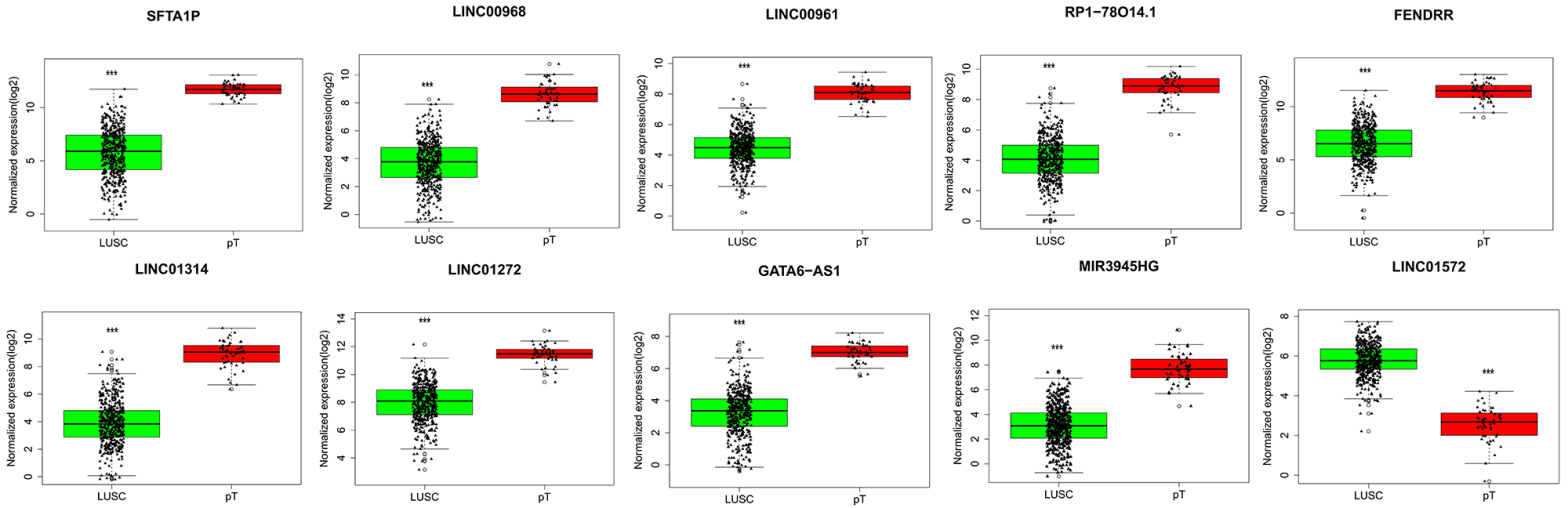
Different expression of the top 10 lncRNAs between LUSC and para-tumorous lung tissues Red column indicates LUSC tissues, and green column indicates lung para-tumorous tissue (pT). The X axis indicates tissue types. The Y axis represents normalized expression of lncRNAs. This figure was drawn by ggplot2 package of R language. *: P<0.05, **: P<0.01, ***: P<0.001.

**Figure 3:**
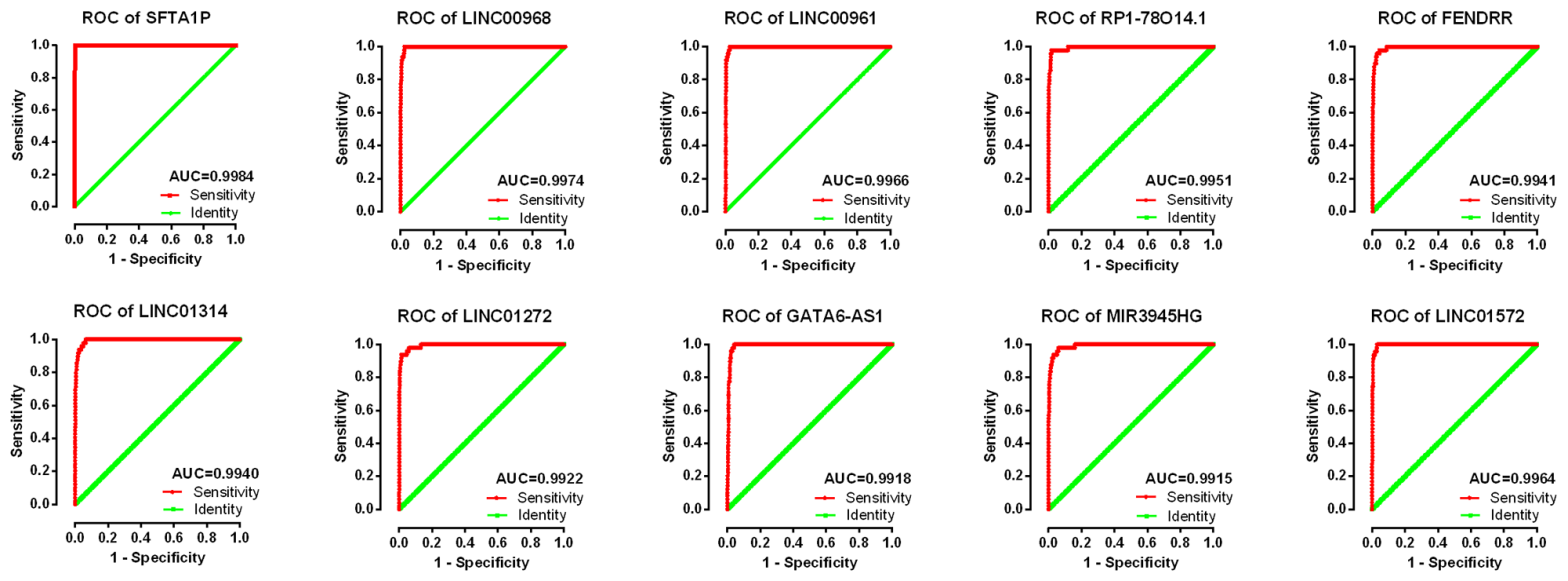
ROC curves of the top 10 lncRNAs sorted by AUC in LUSC Red represents sensitive curve, green indicates identify line. The X axis shows false positive rate, presented as “1-Specificity”. The Y axis indicates true positive rate, shown as “Sensitivity”. These curves were provided by GraphPad Prism 6.

**Figure 4:**
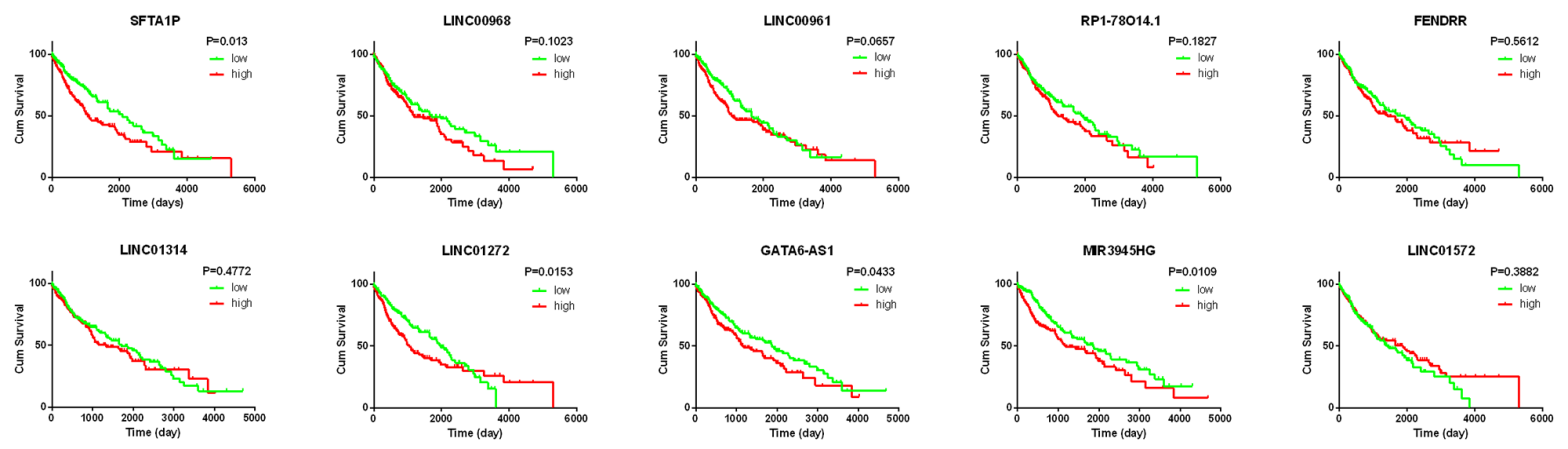
K-M curves of the top 10 lncRNAs in LUSC Red line represents high level of a lncRNA, and green line represents low level. The X axis indicates overall survival time (day), and the Y axis indicates the survival rate. These curves were conducted by GraphPad Prism 6.

**Figure 5:**
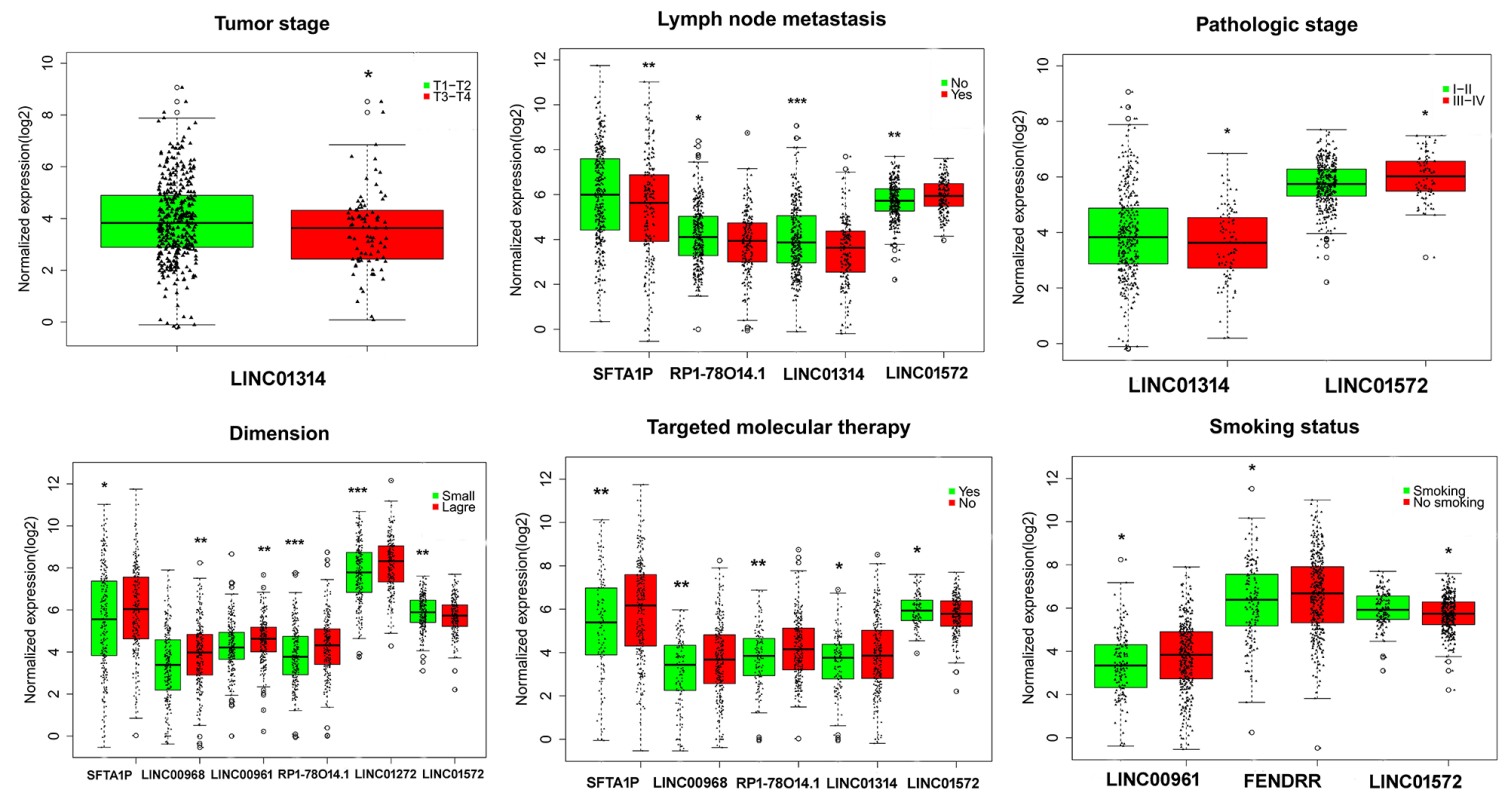
Association between the expression of key lncRNAs and clinicopathological features in LUSC Statistical significance differences of several key lncRNAs were noted in various clinicopathological features: tumor stage (T1/T2 vs. T3/T4), lymph node metastasis (no vs. yes), pathological stage (I/II vs. III/IV), smoking status (no smoking vs. current smoking), targeted molecular therapy (no vs. yes). The X axis indicates different lncRNAs, and the Y axis indicates the normalized expression (log2). The plots were conducted by ggplot2 package of R language. *: P<0.05, **: P<0.01, ***: P<0.001.

**Figure 6:**
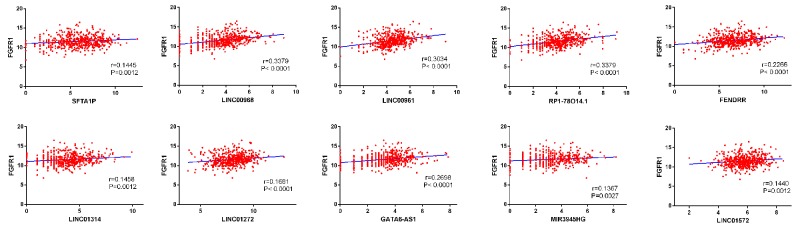
Correlation between FGFR1 expression and lncRNAs in LUSC The expression of these lncRNAs were positively correlated with FGFR1 expression based on TCGA dataset.

The top 10 aberrantly expressed lncRNAs (Table 2) were selected for further analysis, including Surfactant associated 1 (*SFTA1P*), *LINC00968*, *LINC00961*, *LINC01572*, *RP1-78O14.1*, FOXF1 adjacent non-coding developmental regulatory RNA (*FENDRR*), *LINC01314*, *LINC01272*, *GATA6-AS1*, and *MIR3945HG*. The level of *LINC01572* was remarkably higher in the LUSC than that in the para-tumorous lung tissues. On the contrary, the other nine lncRNAs were all obviously downregulated in LUSC tissues (Figure 2). All these 10 aberrantly expressed lncRNAs showed high diagnostic values to distinguish LUSC from non-cancerous lung tissues with AUC all more than 0.99 (Figure 3). Survival analyses showed that *SFTA1P*, *LINC01272*, *GATA6-AS1* and *MIR3945HG* were significantly related to the survival time of LUSC (Figure 4). Further, the multivariate cox analysis showed that SFTA1P might be an independent prognostic indicator for LUSC (P=0.019, Supplementary Table 1). When concerning the relationship between these 10 lncRNAs and the progression of LUSC, several lncRNAs were closely related to some clinical parameters of LUSC (Table 3, Figure 5). Especially, the level of LINC01572 and LINC01314 could distinguish the LUSC patients in early-stage from the advanced-stage. Original data of FGFR1 was extracted from TCGA platform. Significantly positive correlations were noted between FGFR1 and ten-lncRNA (Figure 6).

**Table 2:** Characteristics of top 10 LncRNAs by the AUC size ranking

LncRNA	Ensemble	Location	Regulation	FC	AUC	CI	P-value
SFTA1P	ENSG00000225383	10p14	Down	0.041652365	0.9984	0.996, 1.000	<0.001
LINC00968	ENSG00000246430	8q12.1	Down	0.04726163	0.9974	0.995, 1.000	<0.001
LINC00961	ENSG00000235387	9p13.3	Down	0.100222391	0.9966	0.993, 1.000	<0.001
LINC01572	ENSG00000261008	16q22.2	Up	9.626953056	0.9963	0.992, 1.000	<0.001
RP1-78O14.1	ENSG00000257894	12q21.2	Down	0.054413873	0.9951	0.990, 1.000	<0.001
FENDRR	ENSG00000268388	16q24.1	Down	0.05863219	0.9941	0.989, 0.999	<0.001
LINC01314	ENSG00000259417	15q25.1	Down	0.047958874	0.9940	0.989, 0.999	<0.001
LINC01272	ENSG00000224397	20q13.13	Down	0.122249416	0.9922	0.985, 0.999	<0.001
GATA6-AS1	ENSG00000266010	18q11.2	Down	0.105033789	0.9918	0.985, 0.998	<0.001
MIR3945HG	ENSG00000251230	4q35.1	Down	0.050535778	0.9915	0.983, 0.999	<0.001

FC: fold change; AUC: area under the curve

CI: confidence interval

**Table 3:** Relationship between the expression of the top 10 lncRNAs and clinicopathological factors in LUSC from TCGA

LncRNA\factor	Dimension (small/large)		Smoking (no/yes)		T (T1/2 vs. T3/4)		N (no/yes)		M (no/yes)		Pathological stage (I/II vs III/IV)		Targeted molecular therapy (no/yes)	
	t	P	t	P	t	P	t	P	t	P	t	P	t	P
SFTA1P	-2.236	**0.026**	-1.097	0.273	1.681	0.093	-2.670	**0.008**	1.182	0.238	0.020	0.984	-2.542	**0.011**
LINC00968	-2.752	**0.006**	-2.549	**0.011**	1.138	0.256	-0.269	0.788	0.950	0.343	0.989	0.323	-2.910	**0.044**
LINC00961	-3.169	**0.002**	-1.806	0.072	1.903	0.058	1.635	0.103	0.416	0.678	-0.553	0.581	-0.209	0.835
LINC01572	2.408	**0.016**	2.433	**0.015**	-0.096	0.924	3.012	**0.003**	1.959	0.051	-2.717	**0.007**	2.123	**0.034**
RP1-78O14.1	-3.597	**<0.001**	1.020	0.308	0.087	0.930	-2.250	**0.025**	0.644	0.520	1.137	0.246	-2.634	**0.009**
FENDRR	-1.058	0.290	-1.991	**0.047**	1.812	0.071	-0.588	0.536	0.603	0.547	1.133	0.258	-1.497	0.135
LINC01314	-1.036	0.301	-0.201	0.841	2.066	**0.039**	-3.880	**<0.001**	0.493	0.623	1.991	**0.047**	-2.335	**0.020**
LINC01272	-3.333	**0.001**	0.070	0.994	-0.672	0.502	-1.189	0.235	1.430	0.153	0.131	0.896	-1.367	0.172
GATA6-AS1	0.424	0.672	0.996	0.320	0.343	0.732	-0.623	0.534	-0.336	0.737	0.761	0.447	-1.716	0.087
MIR3945HG	-1.730	0.084	1.161	0.246	-0.118	0.907	-1.580	0.115	-0.517	0.605	1.371	0.171	-1.869	0.062

T: tumor stage; N: lymph node; M: metastasis

### Potential molecular mechanism of the top 10 aberrantly expressed lncRNAs in LUSC

**Figure 7:**
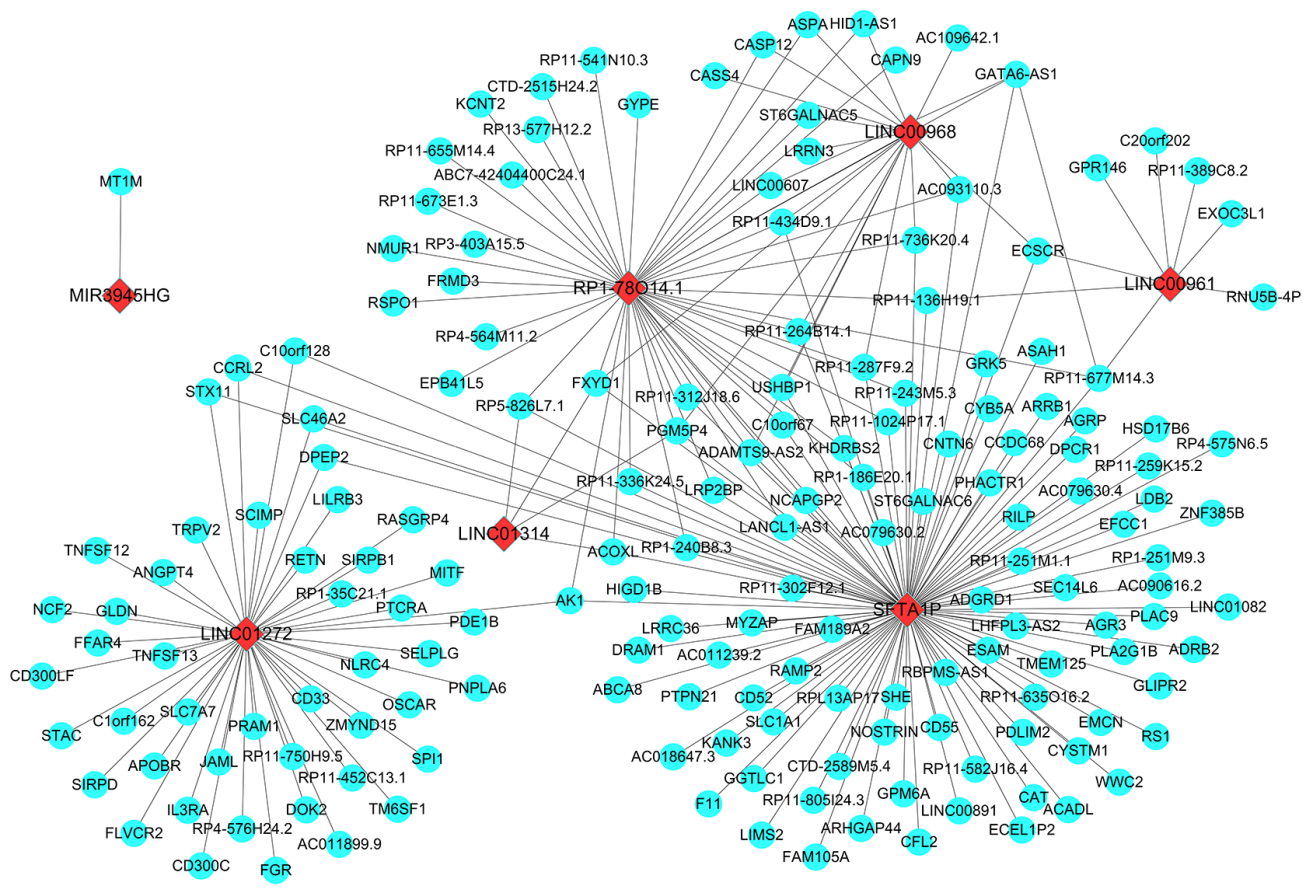
Prospective gene networks of the 10 top differentially expressed lncRNAs To explore the regulation network of the key lncRNAs, the co-expressed genes of those key down-regulated lncRNAs were screened out by WGCNA. Red diamonds showed the key lncRNAs and blue balls are for key lncRNAs co-expressed mRNAs.

The co-expressed genes of all these ten key lncRNAs were determined by the WGCNA. As a result, 120 genes were revealed to be co-expressed with *SFTA1P*, and 47 genes were discovered to have co-expressed relationship with *LINC01272*, as well as the other key lncRNAs (46 genes for *RP1-78O14.1*, 18 for *LINC00968*, 8 for *LINC00961*, 4 for *LINC01314*, and 2 for *GATA6-AS1* and 1 for *MIR3945HG*). Whereas the WGCNA showed no gene being co-expressed with *FENDRR* or *LINC01572* (Figure 7).

**Figure 8:**
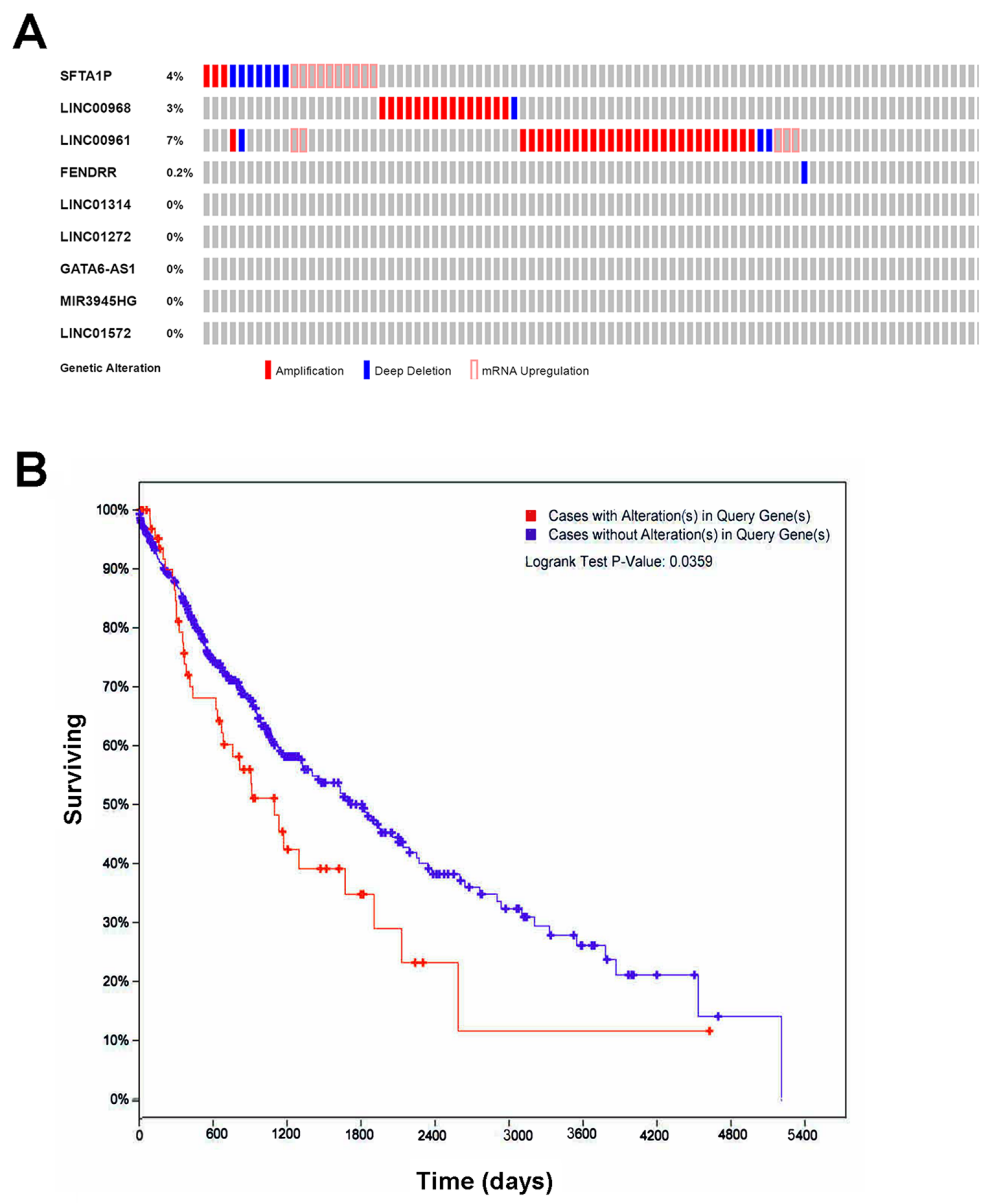
The genetic alterations and their prognostic value of the lncRNAs in LUSC **(A)** Genetic alterations. Red represents amplification, blue represents deep deletion and pink represents mRNA up-regulation. Genetic alterations were found in 69 of 501 LUSC patients (14%). The aberrant expression threshold was defined as z-score ± 2.0 from the TCGA RNA Seq V2 data. This OncoPrint was conducted by cBioPortal. **(B)** K-M curve between groups with alterations and without alterations. Red line represents cases with alterations, and blue line represents cases without. The X axis indicates overall survival time (days), and the Y axis indicates the survival rate. Kaplan-Meier test was performed. These curves were generated by cBioPortal.

The OncoPrint from cBioPortal showed that 14% (69/501) cases with genetic alterations could be obtained (Figure 8A), except *RP1-78O14.1*, whose data were not available in cBioPortal. And only *SFTA1P*, *LINC00968*, *LINC00961*, and *FENDRR* had genetic alterations, including amplification, deep deletion and mRNA upregulation. More interestingly, the cases with genetic alterations had a poorer survival as compared to those without alterations (P=0.0359, Figure 8B). CBioPortal also provided the probable co-occurrence of these top 10 lncRNAs. As Table 4 showed, there was a tendency towards co-occurrence between *SFTA1P* and *LINC00961* in LUSC.

**Table 4:** Results of mutual exclusivity and co-occurrence analysis by cBioPortal.

Gene A	Gene B	P-value	Log odds ratio	Association
SFTA1P	LINC00968	0.515821057	-Infinity	Tendency towards mutual exclusivity
**SFTA1P**	**LINC00961**	**0.04745977**	**1.2549926238226372**	**Tendency towards co-occurrence(Significant)**
SFTA1P	LINC01572	1	Infinity	Tendency towards co-occurrence
SFTA1P	FENDRR	0.96007984	-Infinity	Tendency towards mutual exclusivity
SFTA1P	LINC01314	1	Infinity	Tendency towards co-occurrence
SFTA1P	LINC01272	1	Infinity	Tendency towards co-occurrence
SFTA1P	GATA6-AS1	1	Infinity	Tendency towards co-occurrence
SFTA1P	MIR3945HG	1	Infinity	Tendency towards co-occurrence
LINC00968	LINC00961	0.297586666	-Infinity	Tendency towards mutual exclusivity
LINC00968	LINC01572	1	Infinity	Tendency towards co-occurrence
LINC00968	FENDRR	0.968063872	-Infinity	Tendency towards mutual exclusivity
LINC00968	LINC01314	1	Infinity	Tendency towards co-occurrence
LINC00968	LINC01272	1	Infinity	Tendency towards co-occurrence
LINC00968	GATA6-AS1	1	Infinity	Tendency towards co-occurrence
LINC00968	MIR3945HG	1	Infinity	Tendency towards co-occurrence
LINC00961	LINC01572	1	Infinity	Tendency towards co-occurrence
LINC00961	FENDRR	0.928143713	-Infinity	Tendency towards mutual exclusivity
LINC00961	LINC01314	1	Infinity	Tendency towards co-occurrence
LINC00961	LINC01272	1	Infinity	Tendency towards co-occurrence
LINC00961	GATA6-AS1	1	Infinity	Tendency towards co-occurrence
LINC00961	MIR3945HG	1	Infinity	Tendency towards co-occurrence
LINC01572	FENDRR	1	Infinity	Tendency towards co-occurrence
LINC01572	LINC01314	1	Infinity	Tendency towards co-occurrence
LINC01572	LINC01272	1	Infinity	Tendency towards co-occurrence
LINC01572	GATA6-AS1	1	Infinity	Tendency towards co-occurrence
LINC01572	MIR3945HG	1	Infinity	Tendency towards co-occurrence
FENDRR	LINC01314	1	Infinity	Tendency towards co-occurrence
FENDRR	LINC01272	1	Infinity	Tendency towards co-occurrence
FENDRR	GATA6-AS1	1	Infinity	Tendency towards co-occurrence
FENDRR	MIR3945HG	1	Infinity	Tendency towards co-occurrence
LINC01314	LINC01272	1	Infinity	Tendency towards co-occurrence
LINC01314	GATA6-AS1	1	Infinity	Tendency towards co-occurrence
LINC01314	MIR3945HG	1	Infinity	Tendency towards co-occurrence
LINC01272	GATA6-AS1	1	Infinity	Tendency towards co-occurrence
LINC01272	MIR3945HG	1	Infinity	Tendency towards co-occurrence
GATA6-AS1	MIR3945HG	1	Infinity	Tendency towards co-occurrence

The query contains 5 gene pairs with mutually exclusive alterations (none significant), and 31 gene pairs with co-occurrent alterations (1 significant).

Log odds ratio > 0: Association towards co-occurrence

Log odds ratio <= 0: Association towards mutual exclusivity

P-value < 0.05: Significant association

P-value: Derived from Fisher Exact Test

Log odds ratio: Quantifies how strongly the presence or absence of alterations in gene A are associated with the presence or absence of alterations in gene B in the selected tumors

As a result, the STA1P co-expressed genes were most enriched in lysosome and LINC01272 co-expressed genes were most significantly involved in integral component of membrane. Meanwhile, the most enriched GO terms for mRNAs co-expressed with RP1-78O14.1 was actomyosin structure organization. The result was shown in Table 5. Additionally, we also analyzed the most enriched GO terms within all the mRNAs co-expressed with these lncRNAs. Consequently, plasma membrane was revealed to be the most GO terms and the result was showed in Table 6.

**Table 5:** Significant GO terms based the co-expressed genes with each lncRNA

Category	Term	Count	%	P-value	Fold enrichment	Bonferroni	Benjamini	FDR
**SFTA1P**								
GOTERM_CC_DIRECT	GO:0005764∼lysosome	6	7.89	7.53E-04	8.20	0.06	0.06	0.81
GOTERM_CC_DIRECT	GO:0005886∼plasma membrane	24	31.58	0.002627	1.80	0.21	0.11	2.81
GOTERM_CC_DIRECT	GO:0031225∼anchored component of membrane	4	5.26	0.005591	10.93	0.39	0.15	5.90
GOTERM_CC_DIRECT	GO:0016021∼integral component of membrane	25	32.89	0.022222	1.50	0.86	0.39	21.65
GOTERM_MF_DIRECT	GO:0009055∼electron carrier activity	3	3.95	0.030268	10.82	0.99	0.99	30.54
GOTERM_BP_DIRECT	GO:0016337∼single organismal cell-cell adhesion	3	3.95	0.037736	9.59	1.00	1.00	40.73
GOTERM_BP_DIRECT	GO:0045730∼respiratory burst	2	2.63	0.038785	49.68	1.00	1.00	41.61
GOTERM_CC_DIRECT	GO:0043197∼dendritic spine	3	3.95	0.040391	9.27	0.97	0.52	36.08
GOTERM_MF_DIRECT	GO:0052890∼oxidoreductase activity, acting on the CH-CH group of donors, with a flavin as acceptor	2	2.63	0.044389	43.28	1.00	0.96	41.63
GOTERM_BP_DIRECT	GO:0043149∼stress fiber assembly	2	2.63	0.04462	43.06	1.00	0.99	46.25
GOTERM_MF_DIRECT	GO:0003995∼acyl-CoA dehydrogenase activity	2	2.63	0.047279	40.58	1.00	0.90	43.69
GOTERM_BP_DIRECT	GO:0033539∼fatty acid beta-oxidation using acyl-CoA dehydrogenase	2	2.63	0.053306	35.88	1.00	0.99	52.53
GOTERM_BP_DIRECT	GO:0019370∼leukotriene biosynthetic process	2	2.63	0.059055	32.29	1.00	0.98	56.30
GOTERM_CC_DIRECT	GO:0031674∼I band	2	2.63	0.070735	26.86	1.00	0.66	54.90
GOTERM_BP_DIRECT	GO:0046686∼response to cadmium ion	2	2.63	0.073276	25.83	1.00	0.99	64.47
GOTERM_MF_DIRECT	GO:0004857∼enzyme inhibitor activity	2	2.63	0.086845	21.64	1.00	0.96	65.95
GOTERM_MF_DIRECT	GO:0000062∼fatty-acyl-CoA binding	2	2.63	0.086845	21.64	1.00	0.96	65.95
**LINC01272**								
GOTERM_CC_DIRECT	GO:0016021∼integral component of membrane	24	57.14	0.000105	2.07	0.01	0.01	0.10
GOTERM_BP_DIRECT	GO:0050900∼leukocyte migration	5	11.90	0.000131	18.60	0.03	0.03	0.17
GOTERM_CC_DIRECT	GO:0005886∼plasma membrane	21	50.00	0.000134	2.27	0.01	0.00	0.13
GOTERM_BP_DIRECT	GO:0050776∼regulation of immune response	5	11.90	0.000552	12.75	0.11	0.06	0.70
GOTERM_CC_DIRECT	GO:0005887∼integral component of plasma membrane	10	23.81	0.003022	3.14	0.16	0.05	2.94
GOTERM_BP_DIRECT	GO:0007165∼signal transduction	8	19.05	0.010545	3.13	0.90	0.54	12.62
GOTERM_BP_DIRECT	GO:0007169∼transmembrane receptor protein tyrosine kinase signaling pathway	3	7.14	0.017961	14.18	0.98	0.63	20.60
GOTERM_MF_DIRECT	GO:0005164∼tumor necrosis factor receptor binding	2	4.76	0.058461	32.34	1.00	1.00	48.36
GOTERM_BP_DIRECT	GO:0002376∼immune system process	2	4.76	0.060391	31.30	1.00	0.94	54.75
GOTERM_BP_DIRECT	GO:0045087∼innate immune response	4	9.52	0.063923	4.22	1.00	0.91	56.87
GOTERM_BP_DIRECT	GO:0001525∼angiogenesis	3	7.14	0.082417	6.11	1.00	0.93	66.54
**RP1-78O14.1**								
GOTERM_BP_DIRECT	GO:0031032∼actomyosin structure organization	2	8.33	0.019131	95.68	0.79	0.79	18.65
GOTERM_BP_DIRECT	GO:0006821∼chloride transport	2	8.33	0.028223	64.58	0.90	0.69	26.35
GOTERM_MF_DIRECT	GO:0008092∼cytoskeletal protein binding	2	8.33	0.039095	46.89	0.84	0.84	31.40
GOTERM_CC_DIRECT	GO:0019898∼extrinsic component of membrane	2	8.33	0.065433	27.78	0.87	0.87	43.81
GOTERM_MF_DIRECT	GO:0005200∼structural constituent of cytoskeleton	2	8.33	0.087494	20.46	0.99	0.88	57.91

**Table 6:** Significant GO terms based the all the mRNAs co-expressed with lncRNAs

Category	Term	Count	%	P-Value	Fold enrichment	Bonferroni	Benjamini	FDR
GOTERM_CC_DIRECT	GO:0005886∼plasma membrane	45	34.35115	1.82E-05	1.82569	0.002129	0.002129	0.020817
GOTERM_CC_DIRECT	GO:0016021∼integral component of membrane	52	39.69466	2.27E-05	1.683908	0.002657	0.001329	0.025982
GOTERM_BP_DIRECT	GO:0050900∼leukocyte migration	6	4.580153	0.000705	8.426899	0.309001	0.309001	1.017256
GOTERM_BP_DIRECT	GO:0045730∼respiratory burst	3	2.290076	0.002471	39.5416	0.726545	0.47707	3.523245
GOTERM_CC_DIRECT	GO:0005887∼integral component of plasma membrane	18	13.74046	0.003928	2.126832	0.369021	0.142294	4.397623
GOTERM_BP_DIRECT	GO:0001525∼angiogenesis	6	4.580153	0.00948	4.610232	0.993201	0.810559	12.89588
GOTERM_BP_DIRECT	GO:0031032∼actomyosin structure organization	3	2.290076	0.010552	19.03855	0.996147	0.750853	14.25337
GOTERM_CC_DIRECT	GO:0005764∼lysosome	6	4.580153	0.011147	4.438743	0.730576	0.279542	12.022
GOTERM_MF_DIRECT	GO:0009055∼electron carrier activity	4	3.053435	0.013857	7.897544	0.954853	0.954853	16.29635
GOTERM_MF_DIRECT	GO:0005102∼receptor binding	7	5.343511	0.013998	3.523692	0.956257	0.790851	16.44801
GOTERM_BP_DIRECT	GO:0050776∼regulation of immune response	5	3.816794	0.019727	4.813116	0.999971	0.876075	25.08463
GOTERM_BP_DIRECT	GO:0008277∼regulation of G-protein coupled receptor protein signaling pathway	3	2.290076	0.021302	13.18053	0.999987	0.847489	26.8108
GOTERM_CC_DIRECT	GO:0072557∼IPAF inflammasome complex	2	1.526718	0.029285	66.87706	0.969117	0.50118	28.79674
GOTERM_CC_DIRECT	GO:0031225∼anchored component of membrane	4	3.053435	0.029629	5.918324	0.970371	0.443729	29.08439
GOTERM_MF_DIRECT	GO:0004046∼aminoacylase activity	2	1.526718	0.032953	59.23158	0.999412	0.916227	34.7645
GOTERM_MF_DIRECT	GO:0001665∼alpha-N-acetylgalactosaminide alpha-2,6-sialyltransferase activity	2	1.526718	0.032953	59.23158	0.999412	0.916227	34.7645
GOTERM_BP_DIRECT	GO:0007165∼signal transduction	13	9.923664	0.035075	1.918613	1	0.930938	40.40209
GOTERM_CC_DIRECT	GO:0031256∼leading edge membrane	2	1.526718	0.046447	41.79817	0.996169	0.548389	41.9262
GOTERM_BP_DIRECT	GO:0046470∼phosphatidylcholine metabolic process	2	1.526718	0.056302	34.26939	1	0.977531	56.82828
GOTERM_BP_DIRECT	GO:0032868∼response to insulin	3	2.290076	0.057315	7.672251	1	0.967821	57.49532
GOTERM_MF_DIRECT	GO:0016811∼hydrolase activity, acting on carbon-nitrogen (but not peptide) bonds, in linear amides	2	1.526718	0.059592	32.30813	0.999999	0.966959	54.30836
GOTERM_BP_DIRECT	GO:0009312∼oligosaccharide biosynthetic process	2	1.526718	0.061756	31.15399	1	0.964572	60.3076
GOTERM_BP_DIRECT	GO:0045444∼fat cell differentiation	3	2.290076	0.066642	7.041655	1	0.962572	63.20096
GOTERM_BP_DIRECT	GO:0007171∼activation of transmembrane receptor protein tyrosine kinase activity	2	1.526718	0.06718	28.55782	1	0.952007	63.50671
GOTERM_CC_DIRECT	GO:0005856∼cytoskeleton	6	4.580153	0.069766	2.703924	0.999789	0.652735	56.23642
GOTERM_BP_DIRECT	GO:0007166∼cell surface receptor signaling pathway	5	3.816794	0.07431	3.126769	1	0.955507	67.34839
GOTERM_MF_DIRECT	GO:0052890∼oxidoreductase activity, acting on the CH-CH group of donors, with a flavin as acceptor	2	1.526718	0.080379	23.69263	1	0.975777	65.63741
GOTERM_BP_DIRECT	GO:0070374∼positive regulation of ERK1 and ERK2 cascade	4	3.053435	0.080812	3.916501	1	0.957316	70.51973
GOTERM_CC_DIRECT	GO:0005925∼focal adhesion	6	4.580153	0.083008	2.565616	0.99996	0.675844	62.84951
GOTERM_BP_DIRECT	GO:0043149∼stress fiber assembly	2	1.526718	0.083264	22.84626	1	0.95202	71.63928
GOTERM_MF_DIRECT	GO:0003995∼acyl-CoA dehydrogenase activity	2	1.526718	0.085505	22.21184	1	0.96338	68.00047
GOTERM_BP_DIRECT	GO:0033539∼fatty acid beta-oxidation using acyl-CoA dehydrogenase	2	1.526718	0.099074	19.03855	1	0.967186	77.96049
GOTERM_BP_DIRECT	GO:0001574∼ganglioside biosynthetic process	2	1.526718	0.099074	19.03855	1	0.967186	77.96049

### Validation of the expression and ROC of the eight lncRNAs with GEO data

**Figure 9:**
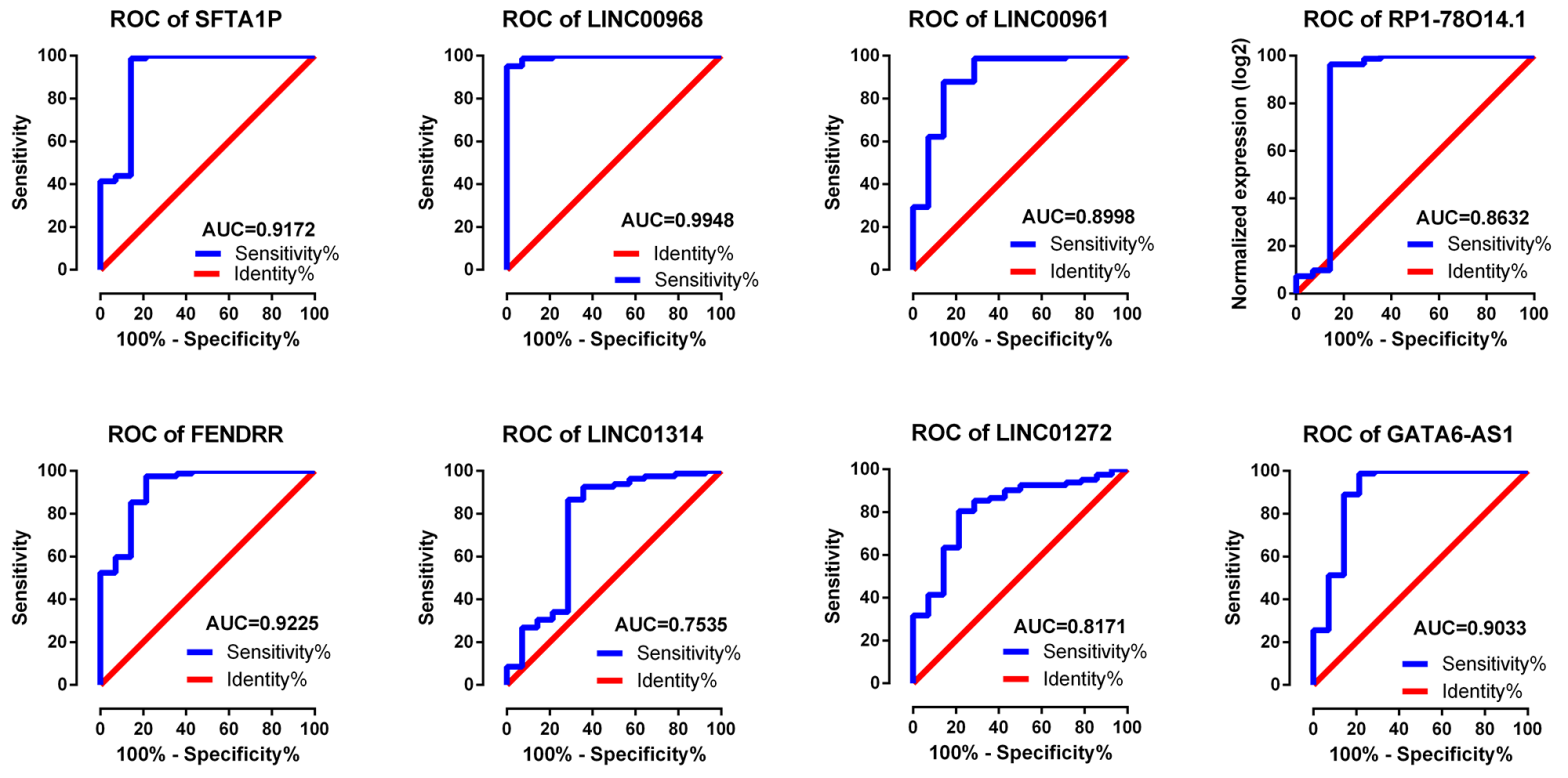
Validation of ROC results of eight lncRNAs in LUSC based on GEO dataset Blue represents sensitive curve, red indicates identify line. The X axis shows false positive rate, presented as “100%- Specificity%”. The Y axis indicates true positive rate, shown as “Sensitivity”. These curves were performed by GraphPad Prism 6.

One study was screened out from GEO datasets (GSE30219). The expression level of eight key lncRNAs, *SFTA1P*, *LINC00968*, *LINC00961*, *RP1-78O14.1*, *FENDRR*, *LINC01314* and *LINC01272*, could be extracted from the dataset, among which the remarkably lower expression of *SFTA1P*, *LINC00968*, *LINC00961*, *RP1-78O14.1*, *FENDRR*, *LINC01314* and *LINC01272* could be observed, while predominantly higher expression of *GATA6-AS1* was found in LUSC tissues (Table 7). The ROC curves of eight lncRNAs all indicated favorable diagnostic value of LUSC (Figure 9).

**Table 7:** Validation of expression and diagnostic value of eight lncRNAs in LUSC based on GEO dataset (GSE30219)

Variable	pT			LUSC			T-test		ROC			
	n	Mean	SD	n	Mean	SD	t	P	AUC	SE	95% CI	P
FENDRR	14	5.214915	0.663845	82	4.295079	0.188372	7.254	<0.0001	0.922	0.0437	0.850 - 0.967	<0.0001
GATA6-AS1	14	5.846385	0.939914	82	5.972000	13.29700	5.972	<0.0001	0.903	0.0613	0.826 - 0.954	<0.0001
LINC00961	14	6.285801	0.370772	82	5.672997	0.255615	7.722	<0.0001	0.900	0.0555	0.822 - 0.952	<0.0001
LINC00968	14	6.824595	1.210060	82	3.556648	0.449696	9.988	<0.0001	0.995	0.0046	0.952 - 1.000	<0.0001
LINC01272	14	4.693669	0.253514	82	4.351741	0.286088	4.574	<0.0001	0.817	0.0619	0.725 - 0.889	<0.0001
LINC01314	14	4.701564	0.272653	82	4.485906	0.155580	2.881	0.0120	0.753	0.0918	0.655 - 0.836	0.0058
RP1-78O14.1	14	5.166360	1.060565	82	3.347113	0.398867	6.342	<0.0001	0.863	0.0883	0.778 - 0.925	<0.0001
SFTA1P	14	7.948137	1.428409	82	5.120226	0.715006	7.254	<0.0001	0.917	0.0561	0.843 - 0.964	<0.0001

pT: para-noncancerous tissue; LUSC: lung squamous cell carcinoma

### Validation based on clinical samples of LUSC

**Figure 10:**
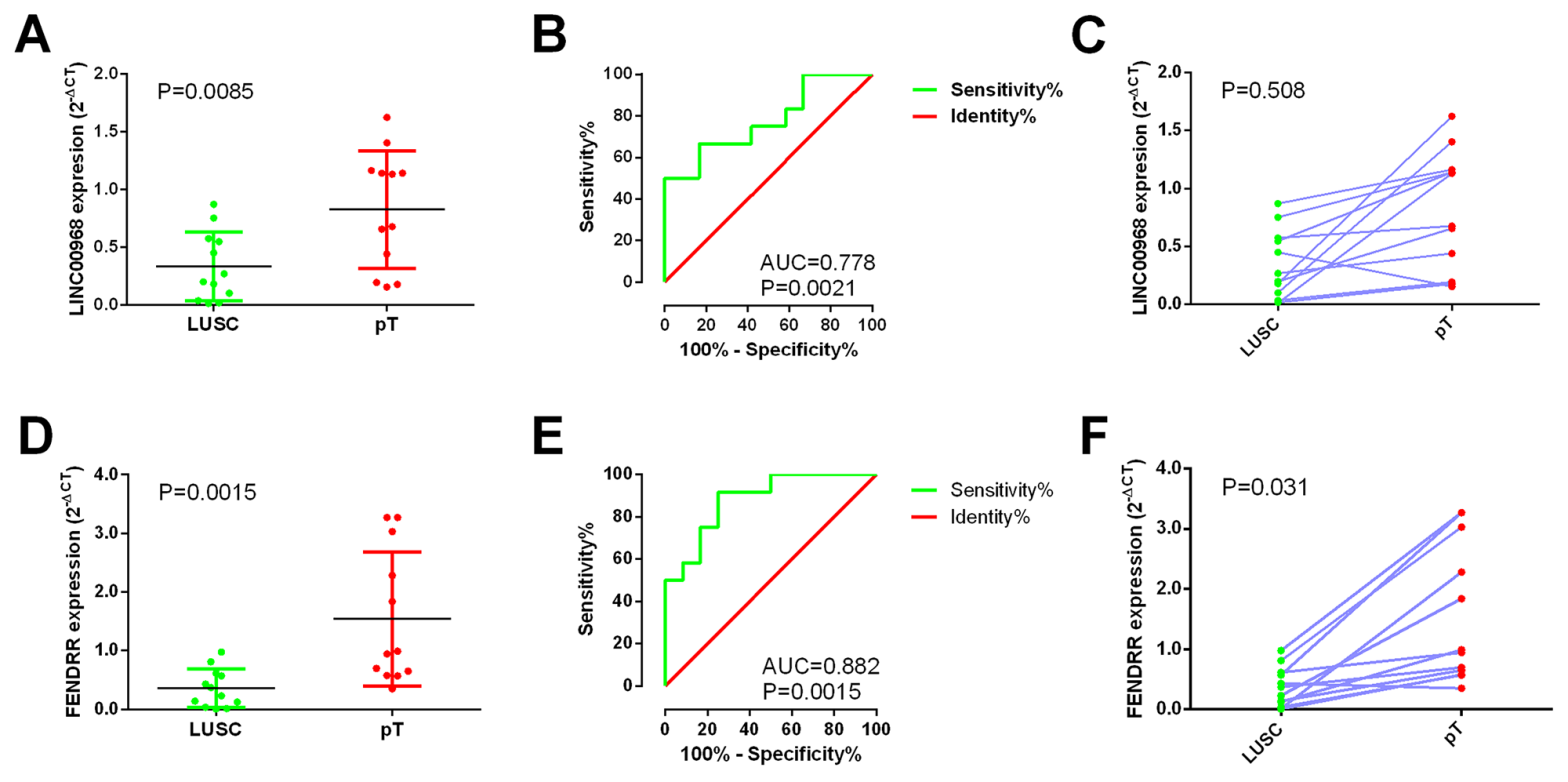
Validation of *LINC00968* and *FENDRR* based on 12 paired clinical samples of LUSC **(A)** The expression of *LINC00968* between para-tumorous lung tissues (pT) and LUSC (RT-qPCR); **(B)** ROC curve of *LINC00968*; **(C)** The correlation of *LINC00968* between para-tumorous lung tissues (pT) and LUSC; **(D)** The expression of *FENDRR* between para-tumorous lung tissues (pT) and LUSC (RT-qPCR); **(E)** ROC curve of *FENDRR*; **(F)** The correlation of *FENDRR* between para-tumorous lung tissues (pT) and LUSC. pT: para-noncancerous tissues.

We performed real time RT-qPCR to confirm the expression of *LINC00968* and *FENDRR* in the 12 paired clinical samples. In these patients, the mean expression level of *LINC00968* was notably lower in LUSC tissues (0.3343±0.08582) than that of non-cancerous lung tissues (0.8258±0.1469; P=0.0085, Figure 10A). Moreover, the AUC of *LINC00968* was 0.778 (P=0.0021, Figure 10B). However, there was no significant correlation between LINC00968 and the tumorigeneses of LUSC (P=0.508, Figure 10C). Meanwhile, the expression trend of *FENDRR* was similar to that of *LINC00968* (P=0.0015, Figure 10D). The AUC of *FENDRR* is 0.882 (P=0.0015, Figure 10E). And we also assessed the relationship between *FENDRR* and the tumorigeneses of LUSC (P=0.031, Figure 10F).

### Further analysis for the key lncRNAs expression in 22 types of cancers based on TCGA

**Figure 11:**
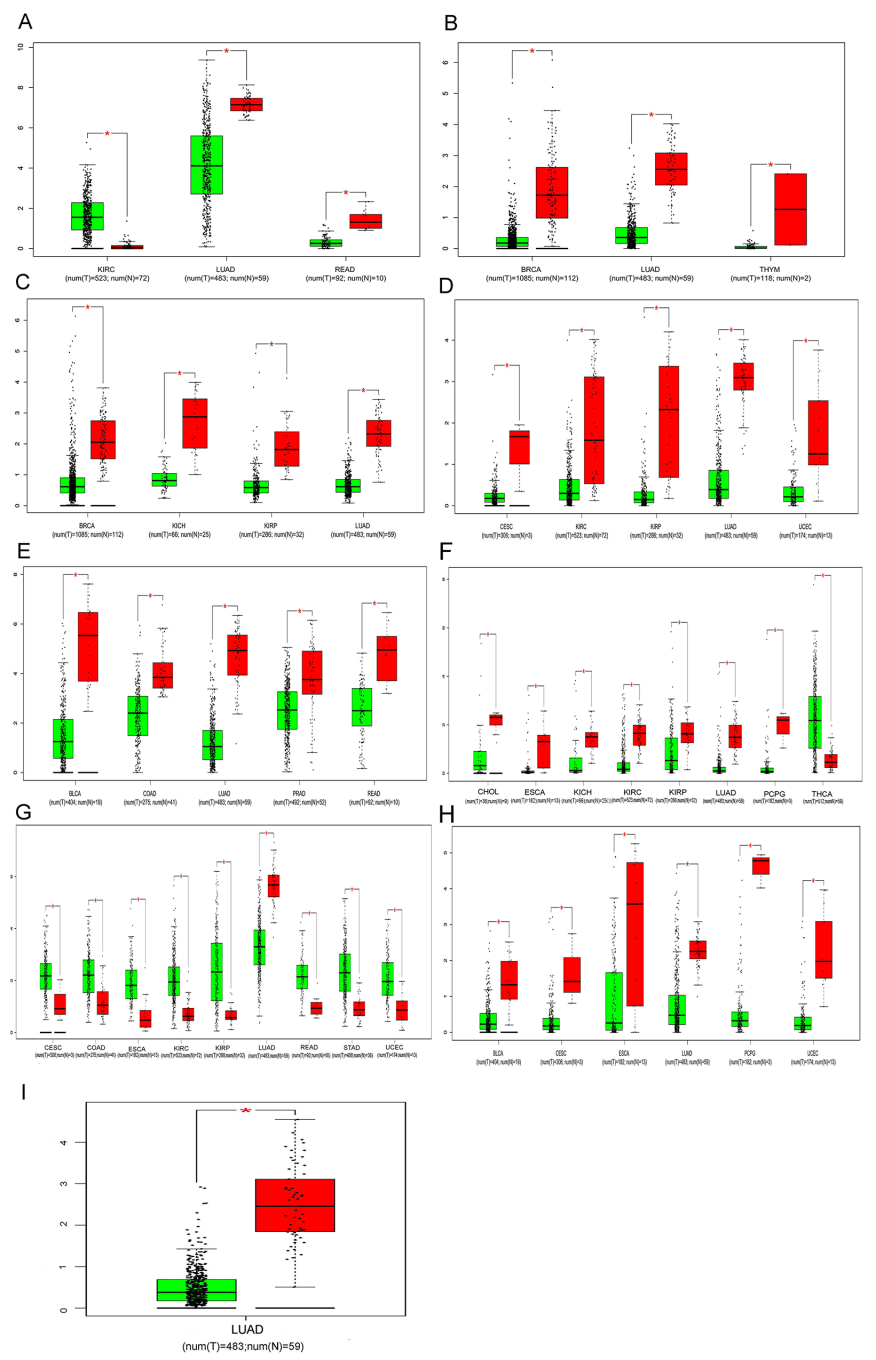
Comparisons of lncRNAs expression between cancer tissues and non-cancerous tissues among 22 types of cancers involved in TCGA based on GEPIA **(A)** SFTA1P; **(B)** LINC00968; **(C)** LINC00961; **(D)** LINC01572; **(E)** RP1-78O14.1; **(F)** FENDRR **(G)** LINC01314; **(H)** LINC01272; **(I)** GATA6-AS1; **(J)** MIR3945HG. Y axis indicates the log2 (TPM + 1) for lncRNA expression. Green bar shows the tumor tissues and red bas indicates the non-cancerous tissues. These figures were derived from GEPIA. *: P<0.05. TPM: Transcripts per Kilobase Million.

Based on the results derived from GEPIA, down-regulation of SFTA1P was found in the lung adenocarcinoma (LUAD) and rectal adenocarcinoma (READ), while the expression of SFTA1P was significantly up-regulated in clear cell kidney carcinoma (KIRC). As shown in the figures, the consistent results were found in breast cancer (BRCA), LUAD and thymoma (THYM), revealing that LINC00968 level was significant lower in these cancers compared with para-noncancerous tissues. consistent with the result in LUSC, the lower expression of LINC00961 was demonstrated in BRCA, kidney chromophobe (KICH), kidney renal papillary cell carcinoma (KIRP) and LUAD. Additionally, lower RP1-78O14.1 expression was also revealed in several types of cancers including cervical squamous cell carcinoma (CESC), KIRC, KIRP and LUAD. Moreover, the significance of FENDRR down-regulation was reached in the bladder urothelial carcinoma (BLCA), colon adenocarcinoma (COAD), LUAD, Prostate adenocarcinoma (PRAD) and READ. Meanwhile, the result also showed the down-regulation of LINC01314 in cholangiocarcinoma (CHOL), esophageal carcinoma (ESCA), KICH, KIRC, KIRP, LUAD and pheochromocytoma and paraganglioma (PCPG), together with the up-regulation in the thyroid carcinoma (THCA). Interestingly, though lower expression of LINC01272 was found in LUAD, the result revealed a significant trend of up-regulation for LINC01272 in CESC, COAD, ESCA, KIRC, KIRP, READ, stomach adenocarcinoma(STAD) and uterine corpus endometrial carcinoma(UCEC). In the support of the result, GATA6-AS1 might act as a tumor suppressor in the several cancers including BLCA, CESC, ESCA, LUAD, pheochromocytoma and paraganglioma (PCPG) and UCEC. Nevertheless, MIR3945HG was only significantly lower in LUAD and there was no significant difference of LINC01572 expression between cancer tissues and para-noncancerous tissues among these 22 cancer types. All the details were presented in the Figure 11, which were derived from GEPIA.

## DISCUSSION

There are marked variances in the aberrant gene profiling and molecular characteristics between LUAD and LUSC, which result in the altered therapeutic regimens administered to the two NSCLC subtypes [24–29]. Development in molecular biology has extended our awareness in decoding a wide scale of genomic unevenness that gradually leads normal lung cells to a cancerous state. In LUAD patients, EGFR-activating somatic mutations in exons 18/19/20/21 modify the sensitivity (namely exon 21 L858R, exon 19 deletion) or resistance (namely exon 20 T790M and/or insertion) to tyrosine kinase inhibitor (TKI) mediated targeted therapeutic strategies. However, as the second most frequent subtype in NSCLC, the treatment possibilities for LUSC remain very inadequate. In the current study, we focused on the aberrantly expressed lncRNAs in LUSC based on TCGA RNA-seq data. Ten lncRNAs with the highest diagnostic value (*SFTA1P*, *LINC00968*, *LINC00961*, *LINC01572*, *RP1-78O14.1*, *FENDRR*, *LINC01314*, *LINC01272*, *GATA6-AS1*, and *MIR3945HG*) were selected for further investigation of their clinical roles in LUSC. Furthermore, these lncRNAs could play essential roles in LUSC via lncRNA-mRNA networks, as well as genetic alterations, including amplification, deep deletion and mRNA upregulation.

EGFR mutations are extremely rare (<5%) in LUSC [30]; nonetheless, other genetic alterations, like overexpression and gene amplification are much common in LUSC, which play pivotal roles in the biological process and disease development of LUSC [31]. This could be explained by the use of cetuximab in the FLEX phase III studies [32], and necitumumab in the SQUIRE study [33, 34]. Except the recently approved molecular target drug nivolumab [35–39], there have been no other recommendations specifically for LUSC as approved by US Food and Drug Administration. The recent molecular advances in lncRNAs could open up a new research area for the clinical setting of LUSC.

Single lncRNA in LUSC has been studied by some groups [40–43]; however, the studies based on high throughput RNA-seq data have been rarely reported. Most recently, Liu et al [22] investigated the altered lncRNAs between LUSC and LUAD. CBioPortal was used to examine lncRNA alteration frequencies, as well as the capacity to evaluate overall survival from TCGA database. In LUSC, 624 lncRNAs were observed to gain alteration rates > 1% and 64 > 10%. Two lncRNAs, including IGF2BP2-AS1 and DGCR5 were related to better overall survival in LUSC. This study [22] focused on the genetic alteration of lncRNAs in LUSC. Similarly, Wei et al [23] also compared the lncRNA transcriptional fingerprints between LUSC and LUAD based on transcriptome analysis with TCGA and GEO. They found that there were 117 dysregulated lncRNAs in LUSC, including 56 up-regulated and 61 down-regulated lncRNAs. Among our top 10 lncRNAs, only *LINC00968* was mentioned in the 117 dysregulated lncRNAs identified by Wei et al [23]. Only 16 cases of paired LUSC tissue samples were examined in the study of Wei et al [23], and this could partially explained the distinction of aberrantly expressed lncRNAs found between Wei et al [23] and our current study.

The top 10 lncRNAs (*SFTA1P*, *LINC00968*, *LINC00961*, *LINC01572*, *RP1-78O14.1*, *FENDRR*, *LINC01314*, *LINC01272*, *GATA6-AS1*, and *MIR3945HG*) had extremely high diagnostic values for LUSC, since the AUCs were all over 0.99. The differential expression levels and diagnostic potency of eight among these 10 lncRNAs could also be confirmed with independent data from GEO, which further supports the findings based on TCGA. We also performed real time RT-qPCR to verify the expression level of two lncRNAs (LINC00968 and FENDRR) with clinical sample in house. Besides, some lncRNAs may also play vital parts in the survival and progression in LUSC, which make them potential novel master regulators for LUSC. Some of these lncRNAs have been reported in other diseases. Among these 10 top aberrantly expressed lncRNAs, only the role and function of FENDRR have been well documented by several studies. *FENDRR* was first identified as a tissue-specific lncRNA, which was a crucial modulator of the growth of heart and body wall in mice [44]. *FENDRR* can bind to Proteasome component 2 (PRC2) and TrxG/MLL complexes to act as a regulator of chromatin signatures that define relevant gene activity [44]. Molecular data also suggests that *FENDRR* plays important part at target regulatory elements via dsDNA/RNA triplex formation, and thus directly raises PRC2 residence at these sites. *FENDRR* can connect epigenetic mechanisms with gene regulatory networks in embryogenesis in the mouse [45]. Furthermore, multiple knockout mouse models also unveil that *FENDRR* is requisite for life and brain development [46]. The clinical role and molecular mechanism of *FENDRR* in cancers also received much attention [47]. Decreased expression of *FENDRR* in infantile hemangioma was detected by both microarray analysis and qPCR [48]. Down-regulation of *FENDRR* was found in gastric cancer and moreover, *FENDRR* was closely related to the poor prognosis in gastric cancer. As for the mechanism, *FENDRR* can modulate the metastasis of gastric cancer cells via influencing fibronectin1 expression [49]. Most recently, high throughput microarray assay and quantitative reverse transcription-polymerase chain reaction (qRT-PCR) were conducted to confirm that *FENDRR* was significantly down-regulated in human Xuanwei lung cancer (XWLC) as compared to that in para normal lung tissues [50]. In the support of this study, we speculated that down-regulation of *FENDRR* might play a vital role in lung cancer based on TCGA dataset and our validation based on a small size of patients by real time RT-qPCR.

*SFTA1P* was first mentioned by a genome-wide association (GWAS) study which investigated the susceptibility genes in the risk for dental caries. SNP rs11256676 in Phenotypes DMFS5_mand_ of Chr. 10p14 was discovered and its function was unknown in 2013 [51]. Interestingly, *SFTA1P* was later reported to be predominately up-regulated in lung adenocarcinoma and one of the most remarkable enriched functions was surfactant homeostasis by array-based transcriptional survey in 2014 [52]. On the contrary, *SFTA1P* was found to be down-regulated in LUSC tissues in the current study, which indicates the distinct role of *SFTA1P* in LUAD and LUSC.

Additionally, two lncRNAs, *MIR3945HG* V1 and *MIR3945HG* V2, were identified as novel candidate diagnostic markers for tuberculosis [53]. But *LINC01314*, *LINC00968*, *LINC00961*, *LINC01572*, *GATA6-AS1*, *RP1-78O14.1* and *LINC01272* are absolutely new lncRNAs, since no publications were available by far. The clinical role of these novel lncRNAs needs further verification in LUSC.

The exact mechanisms of these aberrantly expressed lncRNAs in LUSC remain unknown. An emerging signature tune in the non-coding RNA world goes to the crosstalk between lncRNAs and mRNAs. We then predicted the prospective regulation of lncRNA co-expressed mRNA. Several lncRNAs might exert their functions via co-expressing with mRNA. Even none of WGCNA has been verified in LUSC, it is quite likely to perform in-depth studies to reveal the pathogenesis of LUSC based on aberrantly expressed lncRNAs. Furthermore, the genetic alterations can also regulate the function of certain lncRNA, and thus influence the clinical outcome [54–57]. The roles of lncRNA genetic alterations in LUSC have not been well established. Only several studies explored single lncRNAs and their genetic variants in lung cancer. For instance, among the advanced lung cancer patients, cases with rs3200401 CT and CT + TT genotypes in *MALAT1* had clearly better prognosis than those with the *MALAT1* rs3200401 CC genotype [58]. SNP rs114020893 of *NEXN-AS1* at 1p31.1 might also contribute to lung cancer susceptibility [59].

In the current study, gene amplification, deep deletion and mRNA upregulation were detected in SFTA1P, LINC00968, LINC00961 and FENDRR and these genetic alterations of the lncRNAs showed a close correlation with survival of LUSC. However, the clinical potential of these genetic alterations needs to be confirmed with larger sample size and the exact mechanism of these genetic alterations also required *in vitro* and *in vivo* verification.

Overall, we show a signature of aberrantly expressed lncRNAs in LUSC tissues and the top 10 of them have great clinical value to act as diagnostic biomarkers, and indicators to evaluate the survival and progression of LUSC. However, other precise detecting methods, like real time RT-qPCR or FISH are required to validate the diagnostic potentials of these novel lncRNAs. Also, more in-depth experiments are necessary to explore the underlying mechanism of these lncRNAs in LUSC.

## MATERIALS AND METHODS

### TCGA dataset of LUSC

High throughput data of RNA-Seq diagnosed with LUSC were downloaded from TCGA on November 9, 2016 [22, 23, 60]. These RNA-seq data from Illumina HiSeq RNASeq platform included 504 LUSC and 49 adjacent non-cancerous lung tissues. Since the TCGA data were a community resource project, additional approval by the ethics committee of our hospital was not mandatory. Also, the present study adhered to the TCGA publication guidelines and data access policies.

### Exploration of the aberrantly expressed lncRNAs in LUSC

The RNA-Seq data of LUSC with 60,483 mRNAs covers 7589 lncRNAs, as described by NCBI (https://www.ncbi.nlm.nih.gov/) or Ensembl (http://asia.ensembl .org/). The R language package DESeq [61, 62] was subsequently used for the calculation of aberrantly expressed lncRNAs (adjusted P<0.05 and the absolute log2 fold change >2), respectively. The lncRNAs of which expression was less than 1 in more than 10% of samples were excluded and the expression level of each lncRNA was log2 transformed for the downstream analysis.

### Clinical role of the top 10 aberrantly expressed lncRNAs in LUSC

The receiver operating characteristic (ROC) curve was used to assess the diagnostic effectiveness of all aberrantly expressed lncRNAs in LUSC and the top 10 were then selected for further evaluation. All expression data were presented as the mean ± standard deviation (SD). The different expression levels of the top 10 aberrantly expressed lncRNAs between LUSC and non-cancerous lung tissues, as well as between different clinical groups were assessed by Student’s t test. Pearson correlation test (SPSS Inc., Chicago, IL, USA) was performed for the relationship between FGFR1 and each lncRNA in LUSC. The prognostic roles of these lncRNAs were examined with the Kaplan–Meier method, and the log-rank test was conducted to contradistinguish survival time. The univariate and multivariate cox analyses of these lncRNAs were also performed. A P-value < 0.05 represented statistical significance. The statistical analyses were all carried out by SPSS 22.0.

### Potential molecular mechanism of the top 10 aberrantly expressed lncRNAs in LUSC

To explore the regulation network of the key lncRNAs, the co-expressed genes of those key lncRNAs were screened out by weighted gene co-expression network analysis (WGCNA) [63–65]. Finally, the lncRNA co-expression network was established based on WGCNA and finally visualized by Cytoscape software. Additionally, we also performed the GO analyses for the co-expression genes for six lncRNAs based on the Database for Annotation, Visualization and Integrated Discovery (DAVID, https://david.ncifcrf.gov/).

It could be assumed that the elevated expression of these lncRNAs in LUSC could be caused by genetic alterations, including amplification, deletion, or point mutations. Consequently, cBioPortal was used to summarize the possible genetic alterations for these the top 10 aberrantly expressed lncRNAs in LUSC, which were presented as OncoPrint. The clinical values of the genetic alterations were also evaluated.

### Validation of the aberrant expression and clinical value of lncRNAs in LUSC based on GEO datasets

Data from Gene Expression Omnibus database (GEO, http://www.ncbi.nlm.nih.gov/geo) was used to validate the results from TCGA. Search strategy was as following: (cancer OR carcinoma OR squamous cell carcinoma OR SqCC OR SCC OR tumor OR tumor OR malignanc* OR neoplas*) AND (lung OR pulmonary OR respiratory OR respiration OR aspiration OR bronchi OR bronchioles OR alveoli OR pneumocytes OR “air way”). We only retained the original study that analyzed gene expression profiling between human LUSC tissues and normal control tissues. Independent sample T-test (SPSS 22.0 Inc., Chicago, IL, USA) was used for the statistical analysis of the differentially expressed level of these lncRNAs between LUSC and para-carcinoma lung tissues. The ROC curve analysis was used to validate the diagnostic value of the lncRNAs for LUSC patients based on GEO dataset.

### Validation based on clinical samples of LUSC

To further verify the data from TCGA and GEO, we conducted real time RT-qPCR to detect the level of lncRNA *LINC00968* and *FENDRR* with clinical LUSC samples (n=12) from the First Affiliated Hospital of Guangxi Medical University as previously reported [66–69]. The Ethical Committee of First Affiliated Hospital of Guangxi Medical University, China approved the present study. All participating patients provided informed consent and agreement for the research use of the clinical samples. GAPDH was used as internal reference with the primers as follows: Forward-5’-GCTCTCTGCTCCTCCTGTTC-3’, Reverse-5’-ACGACCAAATCCGTTGACTC-3’. The primers were listed as follows: LINC00968, Forward-5’-CCACTCCTTTAGTCGTTGTGC-3’; Reverse-5’- GGTCCCTCATTCCTATCCC-3’; FENDRR, Forward-5’- TAAAATTGCAGATCCTCCG-3’; Reverse-5’-AACGTTCGCATTGGTTTAGC-3’. Paired-samples t test was performed to compare the difference of lncRNAs between LUSC and non-cancerous lung tissues with SPSS 22.0. ROC curves were used to assess the effect of lncRNAs to discriminate the LUSC from non-cancerous lung tissue.

### Analysis for the expression pattern of the lncRNAs in all tumors involved in TCGA based on GEPIA

We also showed the expression levels of the lncRNAs between cancer tissues and para-noncancerous tissues with the assistance of GEPIA (http://gepia.cancer-pku.cn), which could analyze the RNA sequencing expression data of 23 types of cancers and normal samples from the TCGA according to the standard processing pipeline.

## SUPPLEMENTARY MATERIALS TABLE


