# Supplementary material for: Clinical roles of the aberrantly expressed lncRNAs in lung squamous cell carcinoma: a study based on RNA-sequencing and microarray data mining
Source: Oncotarget. 2017 May 22;8(37):61282–304. doi: 10.18632/oncotarget.18058 (PMC5617423; doi:10.18632/oncotarget.18058)
Supplement: Supplementary file 1 [file oncotarget-08-61282-s001.pdf]

Clinical roles of the aberrantly expressed lncRNAs in lung squamous cell carcinoma: a study based on RNA-sequencing and microarray data mining

SUPPLEMENTARY MATERIALS

Supplementary Table 1: Univariate and multivariate cox analyses for the ten lncRNAs in LUSC

Variables	Univariate				Multivariate			
	P	HR	LL	UL	P	HR	LL	UL
SFTA1P	0.004	1.573	1.154	2.145	0.019	1.550	1.073	2.240
LINC01272	0.019	1.441	1.062	1.954	0.299	1.223	0.836	1.788
GATA6-AS1	0.052	1.356	0.997	1.843	0.767	1.056	0.736	1.516
MIR3945HG	0.014	1.480	1.083	2.022	0.376	1.180	0.818	1.701
LINC00968	0.118	1.276	0.940	1.730				
LINC00961	0.077	1.316	0.971	1.785				
LINC01572	0.356	0.867	0.640	1.174				
RP1_78O14.1	0.207	1.216	0.897	1.647				
FENDRR	0.609	1.082	0.800	1.465				
LINC01314	0.439	1.128	0.831	1.532				
